# Multivitamin-Induced Pharmacobezoar: A Rare Entity of Large Bowel Obstruction

**DOI:** 10.7759/cureus.41688

**Published:** 2023-07-11

**Authors:** Maria del Mar Burgos-Torres, Victor H Molina-Lopez, Natali M Perez Cruz, Charmaine Perez Del Valle, Jose Sorrentino

**Affiliations:** 1 Internal Medicine, VA Caribbean Healthcare System, San Juan, PRI; 2 Cardiology, VA Caribbean Healthcare System, San Juan, PRI; 3 General Surgery, VA Caribbean Helathcare System, San Juan, PRI

**Keywords:** mechanical intestinal obstruction, colonoscopic decompression, large bowel obstruction, pharmacobezoar, alive multivitamins

## Abstract

The term bezoar refers to a foreign object found like a mass of concretion in the gastrointestinal tract that results from an accumulation of undigested material. When the composition of the ingested material is a medication, it is known as a pharmacobezoar. A rare complication from pharmacobezoar is large intestinal obstruction. Here we present the case of a 77-year-old male who presented with progressive abdominal distension, involuntary guarding, and large bowel obstruction. Abdominal imaging studies were remarkable for radiopaque objects of uncertain etiology in the transverse colon and rectal ampulla. The patient underwent colonic decompression by sigmoidoscopy, where the pills were identified by direct visualization. He later underwent endoscopic removal of the pharmacobezoars. A detailed medication review identified the culprit to be multivitamins. This case portrays an unusual etiology of large bowel obstruction. At this moment, no cases have been reported of multivitamins as the culprit of pharmacobezoar with subsequent development of large bowel obstruction.

## Introduction

The term bezoar refers to a foreign object clumped as a mass in the gastrointestinal tract resulting from the accumulation of undigested material. It is referred to as a pharmacobezoar when the composition of the ingested material is a medication [1-2]. Some drugs implicated in the formation of bezoars are aluminum hydroxide, sucralfate, cholestyramine, nifedipine, and psyllium, among others [2]. Additional characteristics of the drug that increase the risk for bezoar formation are extended-release and enteric-coated drug formulations due to decreased solubility [3]. It may happen after intake of combined pharmaceutical preparations of drugs, from overdosing, or even after chronic drug use within the therapeutic doses [2-3]. Patients at increased risk of this complication are those with underlying anatomical variations, previous abdominal surgery, delayed gastric emptying, and underlying chronic constipation. When a pharmacobezoar is present, it rarely presents with intestinal obstruction. It accounts for only 0.4-4% of the cases of large bowel obstruction, with very few cases published [2]. Some case reports have documented large bowel obstruction secondary to pharmacobezoar with extended-release formulations of a drug. Here we present the case of an elderly patient with large bowel obstruction due to ingestion of multivitamins.

## Case presentation

A 77-year-old male with a medical history of hypertension, diabetes mellitus type II, chronic constipation, benign prostatic hyperplasia with chronic indwelling foley, hypothyroidism, and advanced dementia with behavioral disturbances. The patient's home medication list included buspirone, levothyroxine, terazosin, and multivitamins. He was brought to the emergency department (ED) by his caretaker after he presented with general malaise, shortness of breath, and foul-smelling urine. Associated symptoms included two episodes of emesis, described as dark content. Vital signs were pertinent for a fever of 100.4°F and tachycardia of 136 bpm. Physical examination showed a distended abdominal wall but without tenderness or symptoms of peritoneal irritation. Laboratory workup revealed neutrophilic leukocytosis of 13.8x109/uL(normal range: 4.5 to 11.0x109/uL) and normal hemoglobin level. The comprehensive metabolic panel was consistent with acute kidney injury stage I with a creatinine increase from 0.9 mg/dL to 1.4 mg/dL (normal range: 0.7 mg/dL to 1.3 mg/dL) and elevated lactic acid in 3.1 mmol/L (normal range: 0.5 mmol/L to 2.2 mmol/L). Urinalysis showed pyuria and bacteriuria.

**Figure 1:**
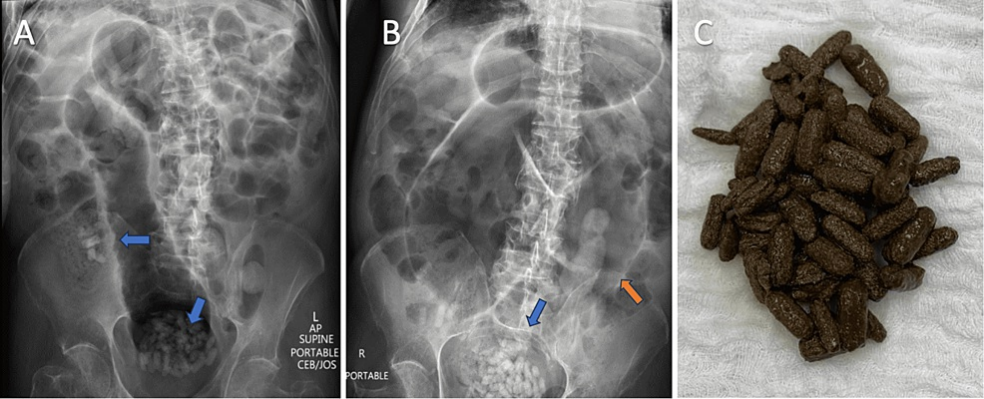
Radio-opaque densities identified on plain radiograph and associated large bowel distention. A. Nonobstructive bowel gas pattern with air distention of the sigmoid colon. Retained radiopaque bodies in the rectal ampulla and right upper quadrant (blue arrows). B. Progression of air distention of the sigmoid colon (orange arrow), fecal impaction, and redemonstration of radio-opaque densities within the rectum (blue arrow). C. Removed pharmacobezoar through colonoscopy.

Due to abdominal distention, a plain radiograph was ordered, which showed a non-obstructive bowel gas pattern, air distention of the sigmoid colon, and the presence of multiple retained radio-opaque foreign bodies in the rectal ampulla and at the right upper quadrant of uncertain etiology (Figure 1A). He was started on intravenous fluids for hydration with close monitoring of his renal function, and gastroenterology service was consulted for recommendations regarding abdominal X-Ray findings. Recommendations included manual desimpaction, soap enemas, polyethylene glycol, stool softener, and serial abdominal X-rays.

Admission was complicated with worsening abdominal distention and obstipation. Despite following gastroenterology service recommendations, a follow-up abdominal X-ray showed progression of air distention in the sigmoid colon, fecal impaction, and redemonstration of multiple radio-opaque densities within the rectum (Figure 1B). On day 5 of admission, physical examination revealed tenderness, and involuntary guarding, accompanied by worsening air distention suggestive of large bowel obstruction in follow-up X-ray. At this point, evaluation by the surgical team was requested due to concerns of development of an acute abdomen. The patient was scheduled for an urgent colonoscopy to perform a colonic decompression due to the impending risk of intestinal perforation. During the colonoscopy, the pharmacobezoar was identified by direct visualization at the level of the rectum.

The team performed a colonoscopic decompression for which a rectal tube was placed. At this moment, the pharmacobezoars were not removed because of the increased risk of intestinal perforation secondary to colonic distention. The follow-up X-ray showed a decrease in the amount of bowel gas and an improvement in large bowel distention. Additional findings included retained feces and an unchanged amount of the radio-opaque foreign bodies. After the procedure, the surgical team recommended serial enemas every 8 hours to manage fecal impaction, but the patient was uncooperative with treatment. Following decompression, the patient had no bowel movements, nor was he passing flatus. Physical examination demonstrated persistent abdominal pain and distention. On day 8 of admission, after addressing colonic distention, the surgery team was able to take the patient to the OR for colonoscopic removal of the pharmacobezoar to relieve the intestinal obstruction. Over 35 pills were removed successfully (Figure 1C), and a follow-up abdominal X-ray reported abundant feces in the transverse colon with improved colonic distention and less numerous radio-opaque densities present. Following the procedure, the patient passed flatus and had regular bowel movements. Physical examination was pertinent for a complete resolution of the previous findings. 

A detailed revision of his medication list was done, and the only drug found to have the same characteristics as the pills removed by colonoscopy were the multivitamins. From the medications listed, it is the only drug with a radiodense appearance on a plain radiograph. This characteristic is secondary to the ingredient potassium chloride found in the vitamins the patient was taking, a trait not shared by any other outpatient medication. It also shared the same morphology and size as the ones removed through colonoscopy, which further supported that the multivitamin was the culprit. Upon discharge to his nursing home, instructions were to continue stool softeners and laxative agents as treatment for constipation but discontinue the use of multivitamins, as these were potentially the cause for the pharmacobezoar. 

## Discussion

Pharmacobezoars are a rare cause of colonic obstruction and can be seen with medications such as fiber-containing laxatives, enteric-coated tablets, and extended-release drugs [2]. Some case reports have also described activated charcoal, aluminum hydroxide, enteric-coated aspirin, and cholestyramine, among other medications, as culprits of pharmacobezoar formation [4]. In most cases, affected patients had an underlying comorbidity such as gastric outlet obstruction, gastrointestinal tract strictures, paralytic ileus, anatomical variations, or prior gastrointestinal surgeries, which predisposed them to this complication [2,4]. Additionally, poor chewing, excessive consumption of fiber supplements, cystic fibrosis, and psychiatric illness, particularly trichotillomania, have been associated [5]. In the case of our patient, he has a medical history of chronic constipation and anatomical variation of a redundant sigmoid colon evidenced by imaging studies, which is known to be a risk factor in the formation of a pharmacobezoar [1]. Identifying these risk factors is essential for the prevention of the formation of a pharmacobezoar, by treating these underlying conditions. In the case of our patient, treatment of his chronic constipation could have decreased the patient's risk of developing this complication.

An accurate diagnosis can be challenging and relies on the bezoar's location. Initial evaluation frequently involves plain radiography or CT scan, which can aid in identifying radiopaque drugs [3]. Sieron DA et al. described in a study performed in 2018 in Switzerland the five most radio-dense pills identified in their patients, which included amiodarone, potassium chloride, bisoprolol, spironolactone, and lisinopril. Other radiopaque medications included multivitamins, iron supplements, and sustained-release medications [6]. This characteristic can facilitate diagnosis when present, but it is important to note that a negative radiograph does not exclude this diagnosis when a radiolucent drug is present [3,6]. It is essential to consider this when the presence of bezoar is high in the differential and radiography is negative. Definitive diagnosis is made by direct visualization via endoscopy. This will depend on the location of the pharmacobezoar. When present in the stomach, esophagus, or colon, endoscopy or colonoscopy is a practical approach [4]. In the case of our patient, the pharmacobezoar was identified initially by plain radiography due to its radiopaque nature. From the patient's medication list, multivitamins were the only drug to have this characteristic secondary to potassium chloride content. A definitive diagnosis was made by direct visualization of the drug with colonoscopy, which was crucial for accurately identifying the offending agent.

When a pharmacobezoar is encountered, a detailed medication reconciliation needs to be performed and the culprit discontinued to avoid the recurrence of intestinal obstruction. This should always include non-prescribed, over-the-counter medications or vitamin supplements that the patient can be taking. Treatment will depend on the bezoar's location, size, and nature. The mainstay for treating uncomplicated intestinal obstruction is conservative management with laxative agents, enemas, manual disimpaction, and close monitoring with serial abdominal imaging. If conservative management fails, a more invasive approach with colonoscopy, endoscopy, or surgery must be taken [3]. In the case of our patient, non-invasive measures failed, with clinical and radiographic progression of intestinal obstruction. Therefore, the surgical team took the patient to the OR for colonoscopic removal of the pharmacobezoar, which was performed without complications and resolved the patient's symptoms successfully. 

## Conclusions

Even though pharmacobezoar is a rare entity of large bowel obstruction, it is important to keep it in our differential. This will lead the therapeutic plan and approach. Additionally, it is vital to recognize patients with comorbidities and anatomical variations that pose an increased risk for the development of this complication because even if it is infrequent, intestinal obstruction could result in a fatal outcome. Diagnosis can be suggested by different imaging modalities, which include a plain radiograph, CT scan, or ultrasound, but confirmation of the diagnosis is done by direct visualization of the bezoar. The therapeutic approach will be dictated by the bezoar's location, size, and nature. This case highlights a rare etiology of bowel obstruction, the importance of prompt diagnosis, the role of medication reconciliation, and colonoscopy as the removal technique.
